# Molecular approaches for enhancing fermented bamboo-derived feed additives: A sustainable nutritional innovation for poultry

**DOI:** 10.1016/j.psj.2025.105766

**Published:** 2025-09-01

**Authors:** Guanlong Li, Aoyu Ji, Emre Yilmaz, Quanxin Wang, Jialu Tong, Xiaolan Liu

**Affiliations:** a Heilongjiang Provincial Key Laboratory of Corn Deep Processing Theory and Technology, College of Food and Bioengineering, Qiqihar University, Qiqihar 161006, China; b Department of Animal Nutrition and Nutritional Diseases, Faculty of Veterinary Medicine, Ataturk University, 25240, Erzurum, Türkiye

**Keywords:** Bamboo, Fermentation, Mutation technology, Sustainable feed additives, Poultry

## Abstract

The function of fermented and non fermented bamboo-derived feed additives in poultry nutrition is critically assessed in this review, with emphasis on the effects on growth performance, immunity, intestinal health, egg and meat quality. Fermented bamboo feeds have become a promising nutritional innovation in poultry production. The use of bamboo leaves and tender shoots in chicken feed is limited due to the presence of lignin and cellulose. Molecular methods, including site-directed mutagenesis and high-throughput screening, could overcome this restriction by enhancing cellulose and lignin degradation, enabling more efficient use of bamboo feed. Microorganisms, such as white-rot fungi (*Pleurotus ostreatus, Ganoderma lucidum, Lentinula edodes*), *Aspergillus niger*, as well as probiotic strains including *Bacillus spp., Lactobacillus fermentum, Lactobacillus rhamnosus, Lactobacillus plantarum*, and *Yarrowia lipolytica*, are commonly utilized during the fermentation process of bamboo-derived feed additives. Microbial fermentation significantly enhances bamboo's nutritional value by increasing its digestibility and lowering its anti-nutritional factors. Furthermore, the presence of bioactive compounds improves the immune response of poultry, thereby reducing the need for antibiotics and fostering sustainable farming practices. In poultry diets, the inclusion level of these supplements may range from 5% to 20%, depending on nutritional demands and production goals. Although the use of these additives as an ingredient in poultry feed has shown promising results, further research is needed to improve the fermentation process and assess the long-term impacts of adding bamboo feeds to poultry diets. This strategy aligns with the worldwide trend toward antibiotic-free and sustainable poultry production systems.

## Introduction

In many developing countries, poultry meat and eggs serve as key sources of animal protein; however, domestic production is often constrained by challenges such as elevated feed costs. Because common feed materials including corn, wheat, soybeans, and groundnuts are not commonly grown locally, commercial chicken farming depends on imported feed supplies. There is significant opportunity to lower production costs and improve the sustainability of poultry systems in these areas by using locally available raw materials as alternative feed resources (Chen et al., 2025; D'Onofrio 2017; Oladayo 2019; Saeed et al., 2024). In contrast to many developing countries, the United States is itself a major global producer of staple feed ingredients such as corn and soybean, ensuring a relatively stable and secure supply for poultry production. However, the U.S. poultry industry is simultaneously confronted with growing regulatory and consumer pressures to reduce antibiotic use, improve animal welfare, and adopt more sustainable production practices. This has intensified interest in natural feed additives, particularly phytogenics and fermentation-based products, that can complement conventional feedstuffs. Some of these alternative feedstuffs contain bioactive substances known as phytogenics, which possess several physiologically active properties beneficial for modern animal production, including antimicrobial, anti-inflammatory, and antioxidant effects. They can also enhance digestive processes by stimulating bile, mucus, and saliva secretion, as well as improving enzyme activity (Applegate et al., 2010; Halvorson et al., 2011; Yao et al., 2023). These physiological effects highlight the potential of phytogenic compounds as feed additives to support animal health and performance; however, their effective and sustainable application will depend not only on a thorough understanding of their chemical composition, practical value, and functional characteristics, but also critically on consumer preferences and expectations (Hashemi and Davoodi, 2011).

Plants and their extracts are frequently utilized in animal nutrition as phytobiotics or phytogenics due to their natural bioactive compounds that promote animal health, enhance performance, and reduce reliance on synthetic additives (Gul et al., 2019; Saeed et al., 2017; Yilmaz and Gul, 2023a; Yilmaz and Gul, 2023b). Among these, bamboo has emerged as a particularly promising plant source in poultry research because of its diverse biological activities, including antioxidant, anti-inflammatory, antimicrobial, and lipid-lowering effects. Bamboo is a fast-growing, woody grass from the *Poaceae* family, comprising more than 1,400 species globally. The total area of bamboo forests worldwide is approximately 31.5 million hectares, accounting for about 1% of the global forest area. However, significant differences among countries in data collection and reporting methods for bamboo resources, which makes it difficult to compare global production Fig.s (Kuehl, 2015). It grows naturally across tropical and subtropical regions of Africa and Asia, with approximately 80% of the world’s bamboo reserves located in China, India, and Myanmar (Lobovikov et al., 2007; Kumar et al., 2014). In contrast, bamboo is not a native or widely cultivated crop in the United States. While small-scale plantings exist in southern states for ornamental, construction, or niche purposes, there is no established large-scale bamboo industry comparable to those in Asia. Nevertheless, given the increasing global emphasis on sustainable agriculture and renewable resources, it is reasonable to anticipate that bamboo cultivation areas may gradually expand in the United States and other countries with suitable agroecological conditions (Vivas et al., 2024). Due to its rapid growth rate, high biomass yield, and ability to thrive in poor soils, bamboo is considered an ecologically sustainable and economically viable plant resource in the USA and other countries (Lobovikov et al., 2007; Kumar et al., 2014). Therefore, throughout history, bamboo has been referred to by various names such as “From Cradle to Coffin Plant,” “Poor Man’s Timber,” “Friend of the People,” “Green Gasoline,” “The Plant with a Thousand Faces,” and “Green Gold” (Borowski et al., 2022). This green gold is an abundant and low-cost resource capable of meeting human needs from birth to death. Currently, there are approximately 3,000 companies worldwide engaged in the production of bamboo-based products, including panels, flooring, pulp, charcoal, edible shoots, and other everyday items (Xuhe, 2003). Bamboo provides raw materials for food, shelter, medicine, construction, wood substitutes, and the paper industry. In addition, it is widely used in the manufacturing of furniture, handicrafts, containers, tool handles, poles, musical instruments, bows and arrows, boats, rafts, fishing rods, and numerous other utilitarian goods (Chongtham et al., 2011). Moreover, it helps in preventing soil erosion and restores degraded land (Verma et al., 2022).

Bamboo has been a particularly popular food source for both people and animals (Chongtham and Bisht, 2020; Satya et al., 2012). Bamboo functions as a natural food source for numerous wild creatures, such as the giant panda, red panda, elephant, giraffe, chimpanzee, and mountain gorilla, which ingest bamboo directly. Numerous animals and birds use the young shoots, leaves, culms, and seeds of diverse bamboo species, with certain species such as the giant panda and golden lemurs relying entirely on bamboo for sustenance (Guo et al., 2020; Xue et al., 2015; Yan et al., 2021a). Various parts of the bamboo plant have been explored in animal nutrition (Bian et al., 2020; Dai et al., 2022; Li et al., 2021; Okano et al., 2009; Wang et al., 2012). Bamboo and its most significant component the leaves are rich in flavonoids, polyphenols, and polysaccharides, which have been shown to exert beneficial effects on growth performance, immune modulation, antioxidant status, and gut health in poultry (Cao et al., 2022; Cao et al., 2023; Okeniyi et al., 2023). In recent years, bamboo leaf extracts have gained attention as functional feed additives, primarily due to their potential to enhance productivity and alleviate oxidative stress in poultry systems (Fathurrahman et al., 2022; Matoq et al., 2024; Negm et al., 2023; Shen et al., 2019a).

Given the growing interest in bamboo as a sustainable feed source—particularly in Asia, where the majority of global bamboo resources are concentrated—this review aims to evaluate the biological effects of different parts of bamboo, the necessity of fermenting bamboo-derived feed additives to enhance their nutritional value, the molecular approaches employed in this fermentation process, and the resulting effects of fermented bamboo-derived feed additives on growth performance and health.

## Bamboo plant fractions

**Fig. 1:**
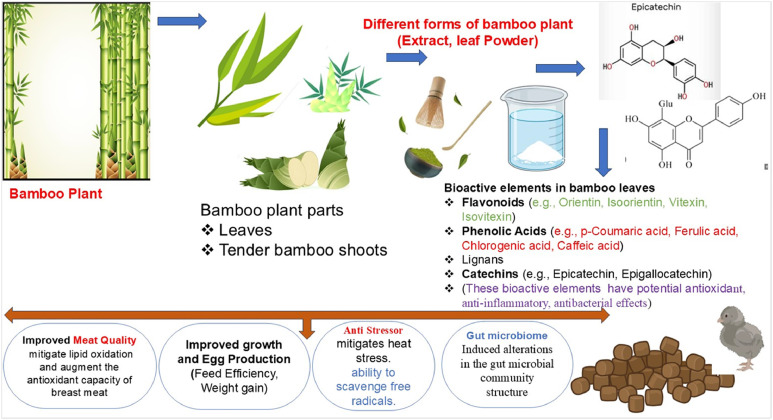
Promising bamboo leaf’s extract effects on poultry production.

Among the various fractions of the bamboo plant—such as leaves, tender shoots, stems, roots, charcoal, and vinegar derivatives—the leaves and young shoots are considered the most nutritionally and functionally significant, particularly in the field of animal nutrition and health (Cacerez et al., 2024; Dai et al., 2022; Kook et al., 2002; Okeniyi et al., 2023; Wang et al., 2012; Yan, et al., 2021b). These components are rich in bioactive compounds such as flavonoids, polyphenols, and dietary fiber, which contribute to numerous physiological benefits when incorporated into animal diets (Akinmoladun, 2022; Anselmo-Moreira et al., 2021; Halvorson et al., 2011; Luo et al., 2019). The specific effects observed in chickens are presented in Figure 1**.**

### Bamboo leaves and leaf extract (or Flavonids)

Bamboo leaves, traditionally used in ethnomedicine across countries such as Japan, China, and Korea to treat conditions like heart disorders, fever, and enteritis, have also been recognized as a valuable non-conventional feed resource for domestic animals such as cattle, sheep, and goats (Bhardwaj et al., 2019; Kitaw et al., 2022; Hayashi et al., 2005; Islam et al., 2024). Thus, bamboo leaves, owing to their favorable nutritional profile, hold promise as a dietary supplement—or even a partial replacement—for conventional forages and crop residues, particularly during periods of feed scarcity such as the dry season (Sasu et al., 2023). Bamboo leaves exhibit considerable variability in their proximate composition depending on species, maturity stage, and environmental conditions. Typically, they contain moderate to high levels of crude protein (approximately 7–17%), relatively low crude fiber (26–34%), and appreciable amounts of essential microminerals (Fe, Mn, Cu, Zn, B and Mo were 144.3, 269.5, 4.23, 27.1, 5.6, and 9.6 mg/kg respectively) and macrominerals such as calcium (1–1.30%), potassium (0.6–1.1%), and magnesium (0.3–0.8%) (Akinmoladun, 2022; Andriarimalala et al., 2019; Chen et al., 2023; Singhal, 2024).

Bamboo leaves yield approximately 11–12% extract, particularly with polysaccharides constituting a major portion of the extractable compounds, when processed via mechanochemical-assisted extraction methods (Zhao et al., 2022). They are a rich source of bioactive compounds such as flavonoids (e.g., kaempferol, rutin, orientin, isoorientin, vitexin, isovitexin, and quercetin) and phenolic acids (e.g., chlorogenic, caffeic, and ferulic acids), as confirmed by HPLC, HPLC-MS, and LC–MS–MS analyses (Akinmoladun, 2022; Luo et al., 2019; Zhang et al., 2018). The phenolic compound content of bamboo varies depending on several factors. For instance, in *Phyllostachys glauca*, a species of bamboo, the total phenolic content was reported to reach 2.278 mg/g, primarily consisting of chlorogenic, neochlorogenic, and cryptochlorogenic acids (Chen et al., 2023). As determined by HPLC analysis, three major phenolic compounds were identified in bamboo leaf extract: chlorogenic acid (1.6 ± 0.1%), caffeic acid (0.1 ± 0.0%), and luteolin‑7‑glucoside (2.8 ± 0.1%) (Bajwa, et al., 2020; Chen, Wang and Liu, 2023; Hu et al., 2000; Rawat et al., 2016). These compounds confer a range of functional properties, which may enhance physiological performance and health in animals when included in diets (Cao et al., 2022; Cao et al., 2023; Shu et al., 2020; Wu et al., 2024). The following sections provide a comprehensive overview of the antioxidant, antimicrobial, prebiotic, and immunomodulatory properties of bamboo leaves in animal feeding.

### Functional properties of bamboo leaves

Bamboo leaves are rich in nutrients and bioactive compounds such as flavonoids, phenolic acids, polysaccharides, phytosterols, and carotenoids, which contribute to their antioxidant, antimicrobial, prebiotic, and immunomodulatory properties (Sasu et al., 2023; Singhal, 2024; Verma et al., 2022). These functional attributes are particularly relevant in poultry production systems aiming to reduce reliance on synthetic additives and antibiotics.

#### *Antioxidant activity*

Bamboo leaves possess significant antioxidant potential, primarily due to their rich content of flavonoids, phenolic acids, vitamin C, and complex polysaccharides (Li et al., 2020; Singhal, 2024; Verma et al., 2022). These bioactive compounds serve as effective free radical scavengers, neutralizing reactive oxygen species and reducing oxidative stress, which prevents lipid peroxidation and protects cellular structures from damage. Key phenolic acids such as p-coumaric acid, ferulic acid, and chlorogenic acid contribute significantly to maintaining redox balance and enhancing cellular defense mechanisms. The antioxidant potential of bamboo leaves is primarily attributed to their high content of flavonoids, particularly orientin, vitexin, and isovitexin. These compounds enhance the defense system by supporting the activation of antioxidant enzymes such as superoxide dismutase (**SOD**), catalase (**CAT**), and glutathione peroxidase (**GSH-Px)**. At the same time, through their strong free radical scavenging and metal-chelating properties (Macwan, et al., 2010), they provide a broad spectrum of antioxidant protection (Luo et al., 2019; Okeniyi et al., 2023). Moreover, some studies suggest that the phenolics in bamboo leaves are relatively stable during feed processing and digestion, which may enhance their practical applicability compared to other phytogenic antioxidants that can degrade under similar conditions (Ma et al., 2020; Shang et al., 2016; Xiao et al., 2022). Another advantage is that bamboo leaves are an inexpensive and sustainable by-product, making them more feasible for large-scale use in poultry nutrition (Islam et al., 2024). Furthermore, advanced extraction techniques such as microwave-assisted extraction have been shown to improve the yield and potency of antioxidant compounds from bamboo, making these bioactives more accessible for practical applications (Milani et al., 2020).

#### *Antimicrobial activity*

Bamboo extracts, particularly those derived from leaves, demonstrate significant broad-spectrum antimicrobial properties (Singh et al., 2010; Tanaka et al., 2013). The gut microbiota communities in chickens play a crucial role in health and function by modulating the immune system, preventing pathogen colonization, and supporting detoxification and nutrient metabolism(Stanley et al., 2014; Wei et al., 2013). There is a strong association between the structure of the gut and the nutrients that are fed to the animal, and it has been hypothesized that the height of the villus can be used to predict the amount of weight growth (Pluske et al., 1996). Bioactive compounds naturally present in bamboo leaf extract —such as stigmasterol, dihydrobrassicasterol, and acetic acid—have been shown to effectively inhibit the growth of various pathogenic and opportunistic microorganisms, including Escherichia coli, Staphylococcus aureus, Lactobacillus spp., and multiple yeast and mold species (Xiao et al., 2020; Yan et al., 2012). Both in vitro and in vivo studies support these findings, underscoring their potential role in modulating gut microbiota. Flavonoids and phenolic compounds isolated from bamboo leaves disrupt bacterial cell membrane permeability, impair intracellular homeostasis, and inhibit microbial proliferation. The antimicrobial efficacy of bamboo leaf polysaccharides varies with sugar residue composition, branching degree, and molecular weight, which may also enhance host defense mechanisms through indirect effects (Jin et al., 2011; Xiao et al., 2020).

#### *Prebiotic potential*

Fibers and polysaccharides derived from bamboo exhibit substantial prebiotic potential by selectively stimulating the growth and metabolic activity of beneficial gut microbiota, particularly *Bifidobacterium adolescentis* and *Bifidobacterium bifidum* (He et al., 2016). These prebiotic compounds act as fermentable substrates, enhancing the proliferation of commensal bacteria and contributing to a balanced intestinal microbial ecosystem. Water-soluble fibers from bamboo are notably effective in modulating gut microbiota composition; their microbial fermentation leads to increased production of short-chain fatty acids (SCFAs), including acetate, propionate, and butyrate (Wu et al., 2020; Yan et al., 2012). These SCFAs are crucial for maintaining epithelial barrier integrity, modulating immune responses, and supporting colonic health through anti-inflammatory and energy-yielding mechanisms. Insoluble fibers, which are especially abundant in bamboo shoots, also play a key role in shaping gut microbial ecology. By increasing fecal bulk and accelerating intestinal transit, they contribute to toxin elimination and overall gut motility. Interestingly, some studies report that insoluble bamboo fibers may have even stronger effects on microbial diversity and SCFAs production than their soluble counterparts (Tanaka et al., 2013; Thomas et al., 2016; Wu et al., 2020). Bamboo-derived polysaccharides, being indigestible in the upper gastrointestinal tract, reach the colon intact, where they interact with resident microbes and modulate host-microbiome metabolic pathways (Porter and Martens, 2017).

#### Immunomodulatory effect

Bamboo leaves possess notable immunomodulatory properties due to their naturally rich composition of flavonoids, phenolic acids, and complex polysaccharides (Porter and Martens, 2017; Shu et al., 2020; Wu et al., 2024). These bioactive compounds contribute to the regulation of immune responses by modulating cytokine secretion, supporting lymphoid organ development, and enhancing the activity of immune effector cells (Di Costanzo et al., 2023; Kim et al., 2019; Shrestha et al., 2018). Regular dietary inclusion of bamboo leaves has been associated with increased proliferation of lymphocytes, improved phagocytic activity of macrophages, and enhanced production of key cytokines such as IL-2 and interferon-γ (Kim et al., 2009; Kumar et al., 2015). In particular, polysaccharides found in the structural components of bamboo leaves are known to interact with gut-associated lymphoid tissue, thereby supporting mucosal immunity and contributing to intestinal immune homeostasis (Porter and Martens, 2017; Shu et al., 2020; Wu et al., 2024; Zhang et al., 2025). These immunological effects manifest as increased antibody responses following vaccination, improved leukocyte profiles, and enhanced resilience to infectious challenges (Kim et al., 2009; Kumar et al., 2015). Furthermore, the anti-inflammatory effects inherent in the phytochemical profile of bamboo may help reduce chronic immune activation and oxidative burden, promoting more efficient immune regulation under production conditions (Guo et al., 2019; Liu et al., 2018; Pang et al., 2023).

### Bamboo-derived other feed additives

Beyond the leaves, various structural parts of the bamboo plant—including the charcoal and vinegar—have attracted growing interest in animal nutrition, particularly in poultry (Rattanawut, 2014; Rattanawut et al., 2021a; Rattanawut et al., 2018a; Rattanawut et al., 2021b; Rattanawut et al., 2017; Rattanawut et al., 2018b; Rattanawut et al., 2019; Yamauchi et al., 2010)**.** Bamboo charcoal (BC) is produced by pyrolyzing bamboo plant material at high temperatures (typically 400–800 °C) under limited oxygen conditions. This controlled thermal decomposition removes volatile compounds and water, yielding a carbon-rich, porous structure with a large surface area and strong adsorption capacity. The bamboo charcoal adsorbs mycotoxins and harmful metabolites in the gastrointestinal tract, reduces substrates available for pathogenic bacteria, binds excess ammonia, and modulates gut microflora. These mechanisms collectively enhance gut integrity, improve nutrient absorption, and promote overall health (Omidiwura et al., 2024; Rattanawut, 2014; Ruttanavut et al., 2009). Its porous structure facilitates adsorption of harmful substances and supports the proliferation of beneficial microbiota, contributing to overall intestinal health. Bamboo vinegar (BV), a liquid by-product generated during charcoal production, contains bioactive compounds such as acetic acid, phenolics, and aromatic esters with antimicrobial and antioxidant properties. The BV suppresses gut pathogens, stimulates beneficial microflora, enhances digestive secretions, improves feed palatability and intake, and reduces microbial spoilage (Ji et al., 2025; Rattanawut et al., 2021a; Rattanawut et al., 2021b).

Numerous studies have investigated the effects of BC, BV, and their combinations (BCV) as dietary supplements in poultry, demonstrating promising results in growth performance, intestinal health, egg quality, and microbial modulation. For instance, Rattanawut (2014) reported that supplementing Betong chickens’ diets with 1% BCV for 16 weeks optimized growth performance, improved intestinal morphology—as evidenced by increased jejunal villus height and area—and reduced fecal pathogenic bacteria such as *Escherichia coli* and *Salmonella spp*. The improvement in growth performance is largely attributed to BC’s ability to adsorb mycotoxins and harmful metabolites in the gastrointestinal tract, which reduces gut stress, prevents pathogen proliferation, and enhances nutrient utilization. In aged laying hens, BCV (1.0–1.5%) improved eggshell thickness, decreased the proportion of damaged eggs, and promoted intestinal villus structure, while lowering ileal pathogen counts (Rattanawut et al., 2017). Dietary BV at 0.4–0.8% also decreased harmful ileal bacteria and improved duodenal villus morphology and eggshell quality, supporting intestinal health (Rattanawut et al., 2018a). Similarly, silicic acid powder containing BV (SPV) enhanced egg production, intestinal villus height, and reduced pathogenic bacteria in laying hens (Rattanawut, Todsadee and Yamauchi, 2018b). In laying ducks, BV at 0.4–0.6% improved production performance and feed efficiency, while decreasing fecal *E. coli* without negatively affecting egg quality (Rattanawut et al., 2019). More comprehensive evaluations of BC, BV, and BCV in laying hens confirmed synergistic effects on egg production, eggshell strength, intestinal morphology, and microbial balance, notably with 0.6% BV promoting villus development and improving feed conversion efficiency (Rattanawut et al., 2021a; Rattanawut et al., 2021b). Additionally, supplementation with bamboo charcoal powder containing vinegar (SB) up to 1.0% improved intestinal histology and tended to enhance egg production in White Leghorn hens (Yamauchi et al., 2010).

The bark, roots, and branches of bamboo, though less frequently studied, contain polyphenolic compounds with immunomodulatory, anti-inflammatory, and antioxidant potential (Akinmoladun, 2022). Extracts from these parts may offer additional health-promoting benefits when formulated into feed additives or natural growth promoters. Several studies have explored other bamboo-derived products and their effects on poultry performance and health. Chen et al. (2011) demonstrated that powdered bamboo leaf extract (**BFRE**) supplementation at 0.3 g/kg (or 0.03%) mitigated the negative effects of cold stress in broiler chickens by preserving body weight gain and stabilizing serum enzyme activities. Dai, Lin, Cheng et al. (2022) reported that micronized bamboo powder (**MBP**) at 1% inclusion improved growth performance, antioxidant status, and cecal microflora diversity in broilers aged 1–22 days. Further research by Dai et al. (2023) showed that replacing 1% maize with MBP during the grower phase enhanced intestinal development and beneficial microbiota without compromising growth. Additionally, Goh et al. (2017) found that bamboo salt (**BS**) supplementation improved feed efficiency in broiler chicks without adversely affecting blood profiles or meat quality.

### Use of bamboo-derived feed additives in poultry nutrition

Increasing regulatory constraints on antibiotic growth promoters (**AGPs**), alongside consumer demand for natural and residue-free poultry products, have heightened the urgency to discover effective alternative feed additives (Saeed et al., 2020; Saeed et al., 2017). The prohibition of AGPs in chicken production has spurred growing attention from both researchers and industry towards natural substitutes like bamboo-based phytobiotics. In contrast to AGPs, these natural growth promoters offer the advantage of improving animal performance without leaving harmful residues in animal-derived products. In this regard, bamboo plant fractions, particularly leaf and leaf extract, have garnered attention as promising components of poultry diets (Kim et al., 2011; Oloruntola et al., 2018; Shu et al., 2020; Wu et al., 2024; Zhou et al., 2024). Their rich content of bioactive compounds—including flavonoids, phenolic acids, polysaccharides, and fiber—provides antioxidant, antimicrobial, immunomodulatory, and prebiotic effects, which improve gut health, immunity, feed efficiency, and growth, especially under stress.

#### Effects on growth performance and welfare in poultry

Bamboo-derived feed additives have gained attention as sustainable and natural alternatives to conventional growth promoters in poultry production. Rich in bioactive phytochemicals such as flavonoids, phenolic acids, polysaccharides, and essential minerals, these additives exhibit antioxidative, immunomodulatory, antimicrobial, and prebiotic properties. These biological activities collectively support gut health, enhance nutrient utilization, and strengthen immune function, thereby contributing to overall bird resilience (Li et al., 2017; Okeniyi et al., 2023; Prihambodo et al., 2020; Wu et al., 2024). Several studies have consistently reported the positive impacts of bamboo leaf flavonoids (**BLF**) and bamboo leaf meal (**BLM**) supplementation on broiler growth performance and physiological welfare. Wu et al. (2024) demonstrated that dietary inclusion of 1000 mg/kg (or 0.1%) BLF significantly increased body weight and average daily gain while reducing feed intake, alongside improving immune responses and antioxidant status. Similarly, Li et al. (2017) observed a 17.6% increase in body weight gain with 2.5 g/kg (or 0.25%) BLF, supporting the growth-promoting capacity of bamboo-derived flavonoids. Okeniyi et al. (2023) extended these findings by showing that up to 2% BLM not only enhanced growth but also alleviated heat stress effects in broilers, indicating the additive’s potential for improving both productivity and welfare under challenging environmental conditions.

Beyond flavonoids and leaf meal, other bamboo derivatives such as powdered BFRE, MBP, BC, and BV have been shown to confer similar benefits. Chen et al. (2011) reported that BFRE supplementation maintained broiler body weight and reduced serum enzyme fluctuations during cold stress, while Dai et al. (2022) and Dai et al. (2023) found that 1% MBP improved weight gain, feed efficiency, and gut morphology. Cacerez et al. (2024) demonstrated that inclusion of activated bamboo charcoal with bentonite clay improved economic returns without compromising growth. Sarker et al. (2016) highlighted the efficacy of 0.2% bamboo vinegar liquid probiotics (**BVLP**) in achieving superior body weight and reduced fat deposition compared to controls and antibiotic groups. Kim et al. (2011) further confirmed that 0.5% BC or leaf supplementation increased terminal body weight in broilers.

Behavioral and physiological welfare aspects have also been addressed in recent research. Matoq et al. (2024) showed that 1–2% BLE supplementation in beak-trimmed ducks reduced abnormal behaviors such as feather pecking and aggression, lowered serum corticosterone levels, and enhanced antioxidant enzyme activities (CAT and SOD). Negm et al. (2023) similarly reported that in their study, bill trimming at 21 d increased serum proinflammatory cytokines (TNF-α, INF-γ, IL-6) and oxidative stress markers (MDA, homocysteine) while decreasing total antioxidant capacity, and that supplementation with BLE attenuated inflammation and oxidative stress and enhanced immune and antioxidant responses, with higher doses being more effective.

#### Effects on immune function and antioxidant status in poultry

Bamboo plant components exhibit significant immunomodulatory and antioxidant properties that are essential for maintaining poultry health, particularly under conditions of oxidative stress. These effects are largely attributed to the presence of high levels of flavonoids, polyphenols, and vitamins such as C and E, which possess potent free radical scavenging and anti-inflammatory activities. Studies by Wu et al. (2024) and Cao et al. (2022) demonstrated that supplementation with BLF significantly enhanced immune function by increasing serum levels of IgA, IgM, and the anti-inflammatory cytokine IL-10, while concurrently reducing levels of pro-inflammatory cytokines such as IL-1β and TNF-α. This immunomodulatory effect is believed to stem from the inhibition of signaling pathways that are triggered by oxidative stress and contribute to immune dysregulation and inflammation. Similarly, Shen et al. (2019a) and Shen et al. (2019b) reported that BLE improved antioxidant status by increasing the activities of key antioxidant enzymes, including total antioxidant capacity, CAT, and GSH-Px. These enhancements were closely associated with the activation of the Nrf2 signaling pathway, a major cellular defense mechanism against oxidative stress. Activation of Nrf2 promotes the expression of a wide range of antioxidant genes, thereby preventing the accumulation of reactive oxygen species and reducing cellular damage.

Several studies have shown that BLE may exert these effects at the mitochondrial level. For instance, Xie et al. (2023) found that BLE supplementation significantly and dose-dependently increased mitochondrial antioxidant enzyme activities and upregulated the expression of mitochondrial biogenesis markers such as SIRT1, Nrf1, and Nrf2. These results suggest that BLE plays an important role not only in cytoplasmic oxidative defense but also in enhancing mitochondrial resilience against oxidative damage. Supporting this evidence, Zhao et al. (2022) reported that BLF supplementation activates the Keap1–Nrf2 signaling pathway, thereby strengthening systemic antioxidant defense mechanisms while also promoting intestinal epithelial integrity—a critical factor in maintaining nutrient absorption and barrier function, particularly under oxidative or inflammatory conditions. Taken together, these findings indicate that bioactive compounds derived from the bamboo plant—especially BLF and BLE—enhance cellular defense through multifaceted antioxidant mechanisms.

#### Effects on gut health and microbiota in poultry

Gut health is a fundamental determinant of nutrient utilization efficiency and disease resistance in poultry, and accumulating evidence suggests that bamboo-derived feed additives can beneficially modulate the gut microbiota. Supplementation with BLF consistently promotes the growth of beneficial bacterial genera including *Lactobacillus, Ruminococcus*, and *Clostridiales*, which are key contributors to SCFAs production and intestinal health (Pei et al., 2020; Shu et al., 2020; Wu et al., 2024). These microbial shifts not only support intestinal ecosystem stability but also enhance metabolic pathways related to fatty acid and amino acid metabolism, as shown in studies with MBP, which increased the abundance of *Firmicutes* while decreasing *Bacteroidetes* (Dai et al., 2022; Dai et al., 2023). Furthermore, MBP supplementation has been associated with elevated intestinal IgA secretion, indicating improved mucosal immunity and barrier function.

The BCV supplementation effectively reduces pathogenic populations of *Escherichia coli* and *Salmonella* spp., while improving intestinal morphology—specifically villus height and surface area—thereby enhancing nutrient absorption and mucosal integrity (Rattanawut, 2014; Rattanawut et al., 2017; Rattanawut et al., 2018a; Rattanawut et al., 2021a; Rattanawut et al., 2021b). Moreover, bamboo charcoal’s detoxifying properties, attributed to its high surface area and adsorption capacity, contribute to reductions in fecal ammonia emissions without disrupting microbial equilibrium, highlighting its dual environmental and gut health benefits (Omidiwura et al., 2024). The BVLP has also demonstrated the potential to enhance gastrointestinal development and metabolic efficiency, evidenced by increased intestinal length and improved meat composition in broilers (Sarker et al. 2016).

Alongside the observed shifts in microbial communities, BLF has shown a capacity to enhance gut barrier integrity. Zhou et al. (2024) reported that dietary BLF elevated the expression of tight junction proteins such as ZO-1, occludin, and claudin-1, thereby reinforcing the intestinal epithelial barrier. This improvement was concomitant with elevated SCFA levels in the cecum, supporting epithelial nourishment and barrier function. The antioxidant and anti-inflammatory properties of BLF likely play a contributory role in these effects. These findings clearly demonstrate that bamboo-derived products—such as BLF, BV, BC, and MBP—provide comprehensive benefits to poultry gut health. They achieve this by reducing harmful gut pathogens, promoting beneficial microbial populations, strengthening the intestinal barrier, and positively regulating both metabolic and immune responses.

#### Effects on meat quality in poultry

The influence of bamboo-derived products on meat quality in poultry is increasingly supported by emerging research, with particular emphasis on improvements in texture, oxidative stability, and nutrient composition. Several studies have converged on the ability of BLF, BC, BV, and their derivatives to modulate meat quality through a combination of antioxidant, metabolic, and structural mechanisms. Shen et al. and Cao et al. (2023) independently demonstrated that supplementation with BLF or BLE at levels ranging between 2 – 3 g/kg (or 0.2–0.3%) enhances broiler meat characteristics by reducing drip loss and shear force, improving pH, and elevating antioxidant enzyme activities such as SOD and GSH-Px. These effects appear to be mediated by activation of the Nrf2 pathway, which regulates oxidative stress responses and promotes meat stability postmortem. Additionally, Cao et al. (2023) identified significant alterations in protein secondary structures and metabolomic profiles, particularly in amino acids, lipids, and organic acids, suggesting that BLF exerts its effects not only via antioxidant pathways but also by reshaping muscle tissue composition and sensory properties like texture and color. This antioxidant-mediated mechanism is consistent with findings by Kim et al. (2011), who noted that BC and bamboo leaf supplementation improved tenderness and increased the unsaturated fatty acid content in broiler meat. These biochemical modifications occurred alongside higher final body weights and better feed conversion ratios, indicating a holistic benefit to both carcass and product quality. Notably, sensory evaluations reported enhanced acceptability in treated groups, reinforcing the consumer-relevant advantages of bamboo supplementation.

Beyond thermoneutral environments, bamboo-derived compounds have demonstrated notable efficacy under environmental stress conditions. For instance, Okeniyi et al. (2023) reported that dietary supplementation with up to 2% BLM in heat-stressed broilers effectively mitigated physiological stress, as indicated by reduced corticosterone levels. Moreover, this supplementation enhanced body weight gain and feed conversion efficiency without eliciting adverse effects on meat quality or organ health. These findings emphasize the potential of bamboo bioactive compounds to stabilize metabolic performance and support resilience during periods of physiological challenge.

#### Effects on egg production and eggshell quality

Numerous studies have investigated the effects of bamboo-derived feed additives on egg production, eggshell quality, and intestinal health, with particular focus on aged laying hens. Rattanawut (2014), Rattanawut et al. (2017), Rattanawut et al. (2021a), and Rattanawut et al. (2021b) consistently demonstrated that supplementation with 0.4–0.8% BCV and BV reduces the incidence of cracked eggs while enhancing eggshell thickness and strength. These improvements are fundamentally attributed to enhanced gut health, as optimization of intestinal morphology increases mineral absorption and reduces intestinal inflammation, thereby contributing to sustained egg production and the maintenance of eggshell quality in older hens.

Considering these findings, the positive effects on gut health not only increase egg production and intestinal villus area but also do so without compromising internal or external egg quality parameters. Notably, the combination of SPV further supports these benefits. Rattanawut, Todsadee and Yamauchi (2018b) demonstrated that dietary supplementation with 2 g/kg (or 0.2%) SPV significantly increased egg production, without adverse effects on internal or external egg quality. In addition, some studies reported improvements in eggshell parameters. For instance, supplementation with bamboo vinegar has been shown to enhance eggshell thickness and eggshell strength, both of which are critical for reducing breakage during handling and storage, thereby ensuring higher economic returns and consumer satisfaction. Similarly, an improved eggshell shape index has also been documented, which contributes to better shell quality and hatchability outcomes in breeding flocks. Furthermore, Wang et al. (2023) reported that bamboo vinegar supplementation improves antioxidant capacity and egg shape index in laying hens under heat stress conditions, thereby indicating the efficacy of bamboo products in supporting egg production even under challenging environmental stressors. The outcomes of different studies on dietary bamboo or different parts ofbamboo supplementation and their beneficial effects across various animal species are summarized in Table 1**.**

**Table 1:** The summarized the differents studies of bambo supplimentation and its beneficial effects in different animal species.

Dietary Supplementation	Animal Species	Main Effects	References
0.3-0.8% BV and 0.3-1.0% BC or their combination	Laying hens	Bamboo supplementation reduced intestine harmful microorganisms and belly obesity in laying hens	Rattanawut, Pimpa, Venkatachalam and Yamauchi (2021a) and Rattanawut, Todsadee [87]
5 g/kg BLM	Broiler	Improved body weight gain	Oloruntola, Agbede, Ayodele and Oloruntola (2018)
0.1 and 1.0 % SB	Aigamo Ducks	Significant intestine histological alteration improved growth performance.	Ruttanavut, Yamauchi, Goto and Erikawa (2009)
0.5, 1.0 and 1.5% SB	White Leghorn hens	Improved production performance	Yamauchi, Ruttanavut and Takenoyama (2010)
0.5, 1.0, and 2% BC	Shark Catfish	bamboo charcoal helped on reduction of ammonia	Quaiyum, et al. (2014)
1, 2.5, and 4% FBP	Swine	It regulates gut microbiota to boost productivity	Sun, et al. (2023)
0.4, 0.8, and 1.2% BC	Tilapia Juvenile	It enhanced growth, blood lipid metabolism, and antioxidant defense	Miao, et al. (2020)
400, 800, and 1600 mg/kg BLF	Broiler	It reduces oxidative damage from repeated heat stress	Yuan, et al. (2025)
20 and 40% BL	Cattle	Bamboo leaves can replace supplements without affecting live weight gain	Altamirano-Gutiérrez, et al. (2023)
200 g BL	Sheep	In ruminant diet, feeding bamboo leaves could help to increase weight gain.	Antwi, et al. (2023)
%1 MBP	Broiler	Feeding micronized bamboo powder promoted gut development, fatty acid metabolism, and fiber degradation bacteria	Dai, Lin, Huang, Yang, Nong, Zuo and et al. (2023)
50,100, and 150 g BL	Boer Goat	Bamboo leaves as a dietary supplement can boost goat farming efficiency and livelihoods in bamboo-rich areas	Zali (2024)
2.0, 4.0, and 6.0 g/kg BLE	Rabbit	Rabbit diet with bamboo leaf extract boosts growth, antioxidants, and cecal microbial composition	Li, et al. (2024)
1.0, 2.0, and 4.0 g/kg BLE	Broiler	Bamboo leaf extract improved broiler small intestine mitochondria metabolism.	Xie, Yu, Yun, Zhang, Shen, Jia, Li, Zhang, Wang and Zhang (2023)

**Abbreviations: BC:** Bamboo charcoal; **BL**: Bamboo leaf; **BLE**: Bamboo leaf extract; **BLF**: Bamboo leaf flavonoids; **FBP**: Fermented bamboo powder; **MBP**: Micronised bamboo powder; **SB**: bamboo vinegar solution.

## Fermentation of bamboo plant fractions

Bamboo constitutes a lignocellulosic biomass characterized by its high content of structural polysaccharides, notably cellulose, hemicellulose, and lignin, which endow the plant material with rigidity and resistance to enzymatic hydrolysis. Among its anatomical fractions, bamboo leaves and shoots are particularly promising as unconventional feed resources in poultry nutrition due to their abundance and phytochemical potential. However, the high concentration of complex, recalcitrant polymers significantly restricts their digestibility and the bioavailability of embedded nutrients (Behera et al., 2014). To overcome these limitations, microbial fermentation has emerged as a pivotal biotechnological strategy aimed at improving the nutritional quality, functional properties, and accessibility of bamboo-derived substrates. This microbial activity enhances nutrient bioavailability, making bamboo more suitable for poultry diets. Improved digestibility decreases the amount of undigested material in the gastrointestinal tract, thereby increasing feed conversion efficiency. Several studies have demonstrated that fermentation with probiotic microorganisms significantly reduces the fiber content in bamboo-derived feed additives (Bajwa et al., 2020; Singhal et al., 2021). Specifically, acid detergent fiber was reported to decrease from 36.7% to 13%, and neutral detergent fiber from 60.6% to 17.3%, in a time-dependent manner. This structural degradation enhances fiber digestibility, resulting in a more bioavailable feed matrix for animals. Furthermore, the fermentation process improves the bioavailability of bioactive compounds such as flavonoids, phenolics, and antioxidants, which contribute to poultry health by reducing oxidative stress and enhancing immune function (Bajwa et al., 2020). Protein content also increases during fermentation, reaching up to 30.4%, with a positive correlation between fermentation duration and protein enrichment. Essential amino acids like lysine and methionine produced during fermentation support protein synthesis and metabolic functions in poultry. Fermentation additionally produces important B-vitamins critical for energy metabolism, immune response, and overall health. The breakdown of cellulose produces SCFAs such as acetic and butyric acid, which provide supplemental energy sources that improve growth and production efficiency in poultry (Liu et al., 2021; Liu et al., 2022).

**Fig. 2:**
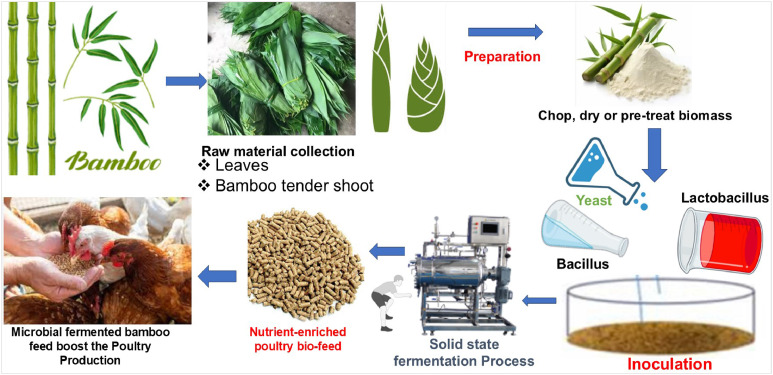
The fermented bamboo as poultry feed ingredient in poultry nutrition.

Fermentation entails a series of microbially driven biochemical conversions, primarily facilitated by lignocellulolytic microorganisms capable of synthesizing specific hydrolytic enzymes (Figure 2). These enzymes catalyze the depolymerization of the plant cell wall matrix—specifically, lignin, cellulose, and hemicellulose—into smaller, more bioavailable molecules, including SCFAs, amino acids, oligosaccharides, and a range of bioactive compounds (Bajwa et al., 2020; Fillat et al., 2017). As a result, the fermentation process not only enhances the digestibility and metabolic utilization of bamboo biomass but also contributes to the enrichment of its nutraceutical profile, rendering it more suitable for monogastric animals such as poultry. The fermentation process typically commences with the mechanical pretreatment of bamboo biomass—such as chopping, grinding, or milling—to increase surface area and facilitate microbial colonization as well as enzymatic accessibility (Liu et al., 2022). Following this preparatory phase, the substrate is inoculated with selected microbial consortia known for their robust lignocellulolytic capacity. Commonly employed strains include white-rot fungi, Aspergillus niger, Bacillus spp., Lactobacillus fermentum, L. rhamnosus, Yarrowia lipolytica, and L. plantarum, all of which play synergistic roles in enzymatic hydrolysis and the bioconversion of structural carbohydrates into fermentable compounds (Huang et al., 2022; Huo et al., 2024; Liu et al., 2022). Through their coordinated enzymatic activities, these microorganisms not only degrade complex polysaccharides but also contribute to the biosynthesis of value-added metabolites, thus enhancing the functionality and nutritional utility of the fermented end-product.

The efficacy of the fermentation process is critically contingent upon the precise regulation of key environmental parameters, namely temperature**,** pH**,** and moisture content**,** each of which exerts a direct influence on microbial growth kinetics, enzymatic expression, and metabolic efficiency. Optimal temperatures are generally maintained between 25°C and 35°C to support the activity of mesophilic fermentative microbes (Behera et al., 2014). The pH is typically controlled within the range of 4.5 to 6.5, a window that favors the catalytic performance of ligninolytic and cellulolytic enzymes while concurrently inhibiting the proliferation of undesirable or competitive microorganisms. Moisture content, a critical determinant of microbial metabolism and enzymatic diffusion, must also be tightly managed to ensure effective substrate solubilization and homogenous microbial activity throughout the fermentation matrix. The duration of fermentation, typically ranging from 5 to 21 days, must be calibrated based on the specific microbial strains employed and the targeted extent of biomass decomposition (Fillat et al., 2017). Shorter durations may result in insufficient cell wall degradation and suboptimal nutrient release, whereas prolonged fermentation may lead to the degradation of valuable nutrients, unfavorable shifts in microbial communities, or spoilage. Thus, identifying and maintaining an optimal fermentation window is essential to maximize the nutritional enhancement of bamboo biomass while ensuring feed safety, microbial stability, and product uniformity.

Failure to maintain these environmental parameters within their ideal thresholds can compromise fermentation outcomes—resulting in incomplete lignocellulose degradation, diminished yields of beneficial metabolites, or contamination by spoilage organisms. Therefore, the standardized control of fermentation conditions remains paramount for achieving consistent, high-quality, and nutritionally optimized bamboo-derived feed products suitable for application in poultry production systems.

## Molecular approaches to fermentation process

The inherent resistance of lignin and cellulose to enzymatic degradation represents a major barrier to the effective biodegradation of bamboo biomass through conventional fermentation methods (Behera et al, 2014). This structural recalcitrance significantly limits the extent to which bamboo-derived materials, particularly leaves and tender shoots, can be directly utilized in monogastric animal nutrition. To address these constraints and enhance the efficiency of biomass conversion, molecular biology-based strategies have gained increasing attention in recent years.

**Fig. 3:**
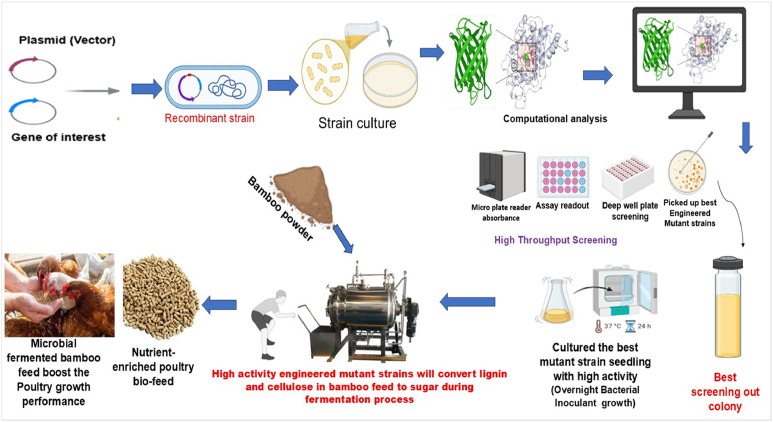
Schematic representation of Molecular techniques used to engineer bacterial strains.

Among the most promising molecular approaches are Site-Directed Mutagenesis (**SDM**) and High-Throughput Screening (**HTS**), enabling targeted manipulation and rapid evaluation of microbial enzymes involved in lignocellulosic degradation. These techniques have been employed to improve the catalytic efficiency and substrate specificity of key ligninolytic enzymes, thereby facilitating the more effective breakdown of bamboo’s structural polymers. Of particular interest are Manganese peroxidase (**MnP**) and Lignin peroxidase (**LiP**), extracellular enzymes central roles in lignin depolymerization, with considerable potential for applications in both agricultural waste valorization and feed fermentation applications. Molecular engineering, broadly defined as the rational design and modification of biological macromolecules at the genetic and protein levels, facilitates the development of microbial strains with enhanced functional traits (Ryu and Nam, 2000; Shi et al., 2025). Such advancements have expanded the toolkit for microbial strain improvement, enabling more efficient bioconversion of recalcitrant plant materials. A summary of commonly applied molecular strategies in bacterial strain engineering is shown in Figure 3**.**

### Site-directed mutagenesis

The site-directed mutagenesis (**SDM**), leveraging the PCR, is a precise and powerful molecular biology technique employed to introduce targeted mutations into specific DNA sequences. This method enables detailed investigation into how alterations in nucleotide sequences influence protein structure, catalytic function, and substrate affinity (Zhang et al., 2000). Owing to its precision and efficiency, SDM has become a widely adopted tool for generating focused mutation libraries rapidly, often obviating the necessity for extensive high-throughput screening. This approach is particularly valuable in engineering enzymes, such as bacterial lignin peroxidases, to enhance catalytic activity, substrate specificity, or other functional attributes (Kardashliev et al., 2014).

Although lignin is a rich source of aromatic and high-value compounds, its application remains limited due to its chemically inert and recalcitrant nature. White-rot basidiomycetes possess the unique ability to degrade lignin through the secretion of a suite of extracellular enzymes, including LiP, MnP, versatile peroxidase, laccase, and associated auxiliary enzymes. Among these, LiP is an oxidoreductase that catalyzes the oxidative cleavage of lignin and its derivatives in the presence of H₂O₂, demonstrating considerable potential across diverse sectors such as second-generation biofuels, cosmetics, the food industry, and bioleaching applications (Biko et al., 2020; Edwards et al., 1993). For instance, in *Pseudomonas putida* KT2440, periplasmic expression of Dyp1B peroxidase from *Pseudomonas fluorescens* and its site-directed mutants have been shown to significantly enhance the degradation of polymeric lignin (Ehibhatiomhan et al., 2023). Additionally, Ambert-Balay et al. (1998) demonstrated that SDM increased the binding affinity of *Phanerochaete chrysosporium* lignin peroxidase for veratryl alcohol, identifying two critical substrate interaction sites via SDM that contributed to the enzyme’s augmented activity (Doyle et al., 1998).

### Directed evolution and high-throughput screening

Directed Evolution (**DE**) is a powerful technique widely employed in molecular biology and genetic engineering to improve or introduce novel functional properties in proteins by mimicking natural selection under laboratory conditions (Yoav et al., 2019). The DE finds applications across various fields including biotechnology, pharmaceutical development, and industrial enzyme production. The process involves the diversification of a target biomolecule through random mutagenesis methods such as error-prone PCR or DNA shuffling, generating extensive mutant libraries (Hossain et al., 2016). For instance, a mutant variant of β-glucosidase Ks5A7, produced via error-prone PCR, exhibited 1.5-fold higher specific activity and enhanced glucose tolerance compared to the wild-type enzyme (Cao et al., 2018). The selection phase of DE consists of iterative rounds of identifying mutants with desirable traits, amplifying their genes, and progressively improving performance until superior mutants are isolated. This approach has been effectively utilized for the development of novel genetic elements for synthetic biology, enzyme optimization, targeted drug delivery protein design, and enhancement of biomolecular functions (Cobb et al., 2013). Notably, fungi have employed DE to evolve ligninolytic enzymes with improved pH tolerance and storage stability (Lei et al., 2023).

The HTS technologies enable rapid and efficient evaluation of mutants. These technologies include single-cell screening on agar plates (Gantz et al., 2023), 96-well microplate assays (Ryu et al., 2008), flow cytometry-based sorting (Scheele et al., 2024), and droplet microfluidics (Oyobiki et al., 2014). The HTS has substantially overcome the limitations of previous screening methods by significantly accelerating the speed and throughput of mutant identification (Beneyton and Rossignol 2021; Ye et al., 2018). For example, Miyazaki‐Imamura et al. (2003) developed a 384-well plate screening platform capable of simultaneously evaluating over 10^4^ mutants, successfully identifying MnP variants tolerant to H₂O₂ with ninefold enhanced stability relative to wild-type enzymes, illustrating the potential of HTS to enhance enzyme performance. Moreover, advancements in protein immobilization techniques combined with flow cytometry have further augmented HTS capabilities. Kojima et al. (2016) employed single-stranded Cro inhibitor tags to immobilize target proteins—such as transglutaminase 2 and MnP—onto microspheres, facilitating functional analyses via flow cytometry. MnP-scCro conjugated microspheres exhibited a 2.5-fold increase in signal compared to controls, confirming retention of enzymatic activity. These immobilization strategies are critical for assessing protein activity, interactions, and ligand binding within HTS workflows. Although relatively few studies have applied HTS following MnP immobilization, future directions include enhancing MnP-directed evolution by integrating phage display and yeast surface display technologies, followed by high-affinity protein screening using flow cytometry (Zeko-Pivač et al., 2022).

### Automated continuous evolution

The Automated Continuous Evolution (**ACE**) system represents an innovative biotechnological approach that enables genes to autonomously and continuously acquire desired functions through multiple mutational adaptation pathways (D'Oelsnitz and Ellington, 2018). By providing an accelerated and controlled simulation of natural selection in vitro, this system promotes increased genetic diversity and rapid emergence of functional mutations. ACE technology offers significant advantages for the optimization, stabilization, or acquisition of novel functions in target proteins or enzymes. When integrated with HTS methodologies, ACE systems facilitate the rapid and efficient identification of superior variants from millions of mutants. This integration substantially accelerates the development and optimization of enzymes and microbial strains in biotechnological applications, particularly in complex, multi-step biochemical processes such as bamboo fermentation. Consequently, it contributes to enhanced production efficiency and product quality. These advancements provide innovative solutions with broad implications for both industrial biotechnology and sustainable agricultural practices (Markel et al., 2020; Zhong et al., 2020).

## Fermented bamboo plant fractions

In recent years, the use of fermented bamboo powder (**FBP**) in poultry nutrition has demonstrated considerable potential for enhancing growth performance, improving intestinal health, and strengthening the immune system. Although raw bamboo and its leaves contain antinutritional compounds that limit their direct use in diets, fermentation effectively mitigates these negative effects and significantly increases nutrient bioavailability. Rahmani Malyar et al. (2024a) and Rahmani Malyar et al. (2024b) demonstrated that dietary supplementation with FBP markedly improved growth performance in dwarf yellow-feathered broilers, an effect supported by enhancements in intestinal morphology and increased levels of gut hormones. Furthermore, the observed upregulation of odorant receptor expression suggests that FBP activates chemosensory pathways within the gut, thereby positively influencing digestive processes. Similarly, Rahmani Malyar et al. (2025) reported that FBP supplementation elevated digestive enzyme activities and antioxidant defense mechanisms while reducing oxidative stress markers and promoting significant improvements in intestinal structure. These findings indicate that FBP optimizes gut function, nutrient absorption, and overall health status. Additionally, Shoura et al. (2025) found that FBP not only enhanced growth performance but also improved blood biochemical parameters, increased antioxidant enzyme activities, and upregulated nutrient transporter gene expression, collectively supporting broiler health through multiple physiological pathways. Previous studies indicate that fermented bamboo powder not only enhances growth-promoting hormone secretion and strengthens immune responses but also improves intestinal morphology, thereby facilitating more efficient nutrient utilization. Additionally, the upregulation of antioxidant capacity and the attenuation of inflammatory stress emerge as key factors positively influencing animal welfare and productive performance.

## Conclusion

The present review highlights both the opportunities and the limitations associated with the use of bamboo-derived products in poultry nutrition. At present, no published data exist from the United States or other developed markets, which represents a critical barrier to their potential adoption. In such high-regulation environments, the Food and Drug Administration (FDA) and the Association of American Feed Control Officials (AAFCO) play a decisive role in approving novel feed additives, determining permissible inclusion levels, and defining labeling requirements. As the FDA framework is widely regarded as a global benchmark, U.S. approval would likely exert a strong influence on the international acceptance of bamboo-based ingredients. Consequently, future research should extend beyond Asian production systems and incorporate pilot-scale trials in developed countries, addressing regulatory compliance, supply chain feasibility, and cost–benefit considerations.

Bamboo leaves and their fermented derivatives are rich in flavonoids, phenolic acids, and antioxidants, conferring nutritional and functional benefits such as improved intestinal health, enhanced protein quality, and greater mineral bioavailability. Fermentation represents a promising biotechnological strategy to overcome the limitations of raw bamboo’s lignocellulosic structure. Nevertheless, significant challenges remain, including the lack of standardization in fermentation protocols, incomplete knowledge of underlying molecular mechanisms, and limited dose–response data across poultry species and production stages. Therefore, while fermented bamboo products exhibit substantial potential as functional feed additives, the current body of evidence is fragmented and insufficient for broad application. Future research should prioritize standardized fermentation methodologies, mechanistic investigations, and well-designed trials under diverse production systems, complemented by systematic reviews and meta-analyses to consolidate findings. Such efforts are essential to substantiate the efficacy, safety, and practicality of bamboo-derived feed additives in modern poultry production.

## CRediT authorship contribution statement

**Guanlong Li:** Writing – original draft. **Aoyu Ji:** Writing – review & editing. **Emre Yilmaz:** Writing – review & editing. **Quanxin Wang:** Data curation. **Jialu Tong:** Data curation. **Xiaolan Liu:** Data curation.

## Disclosures

**all authors** Guanlong Li ^a, *^, Aoyu Ji ^a^, Emre Yilmaz^b^, Quanxin Wang ^a^, Jialu Tong ^a^, Xiaolan Liu ^a, *^; approved of its submission to Poultry Science and no any conflict of interest
